# Calcification of the Anterior Acromial Insertion of the Deltoid Muscle

**DOI:** 10.1155/2020/8895801

**Published:** 2020-10-10

**Authors:** Kiminori Yukata, Ashish Suthar, Yutaka Suetomi, Kazuhiro Yamazaki, Kazuteru Doi, Hiroshi Fujii

**Affiliations:** Department of Orthopedic Surgery, Ogori Daiichi General Hospital, Yamaguchi, Japan

## Abstract

A 45-year-old man presented with severe left shoulder pain that was not associated with trauma. Plain radiography with the arm in an elevated position and ultrasonography demonstrated calcium deposits at the anterior acromial insertion site of left deltoid muscle. Conservative management could successfully relieve pain. At the 3-year follow-up, the calcification completely disappeared. To the best of our knowledge, calcium deposits at the acromial insertion site of the deltoid have not been reported in the literature. Clinicians who suspect calcific tendinitis but do not observe calcification around the rotator cuff should carefully palpate and examine other sites, such as the deltoid origin, and use ultrasonography or radiography.

## 1. Introduction

Calcific tendinopathy is a common cause of shoulder pain [1, 2]. Calcium deposits, hydroxyapatite crystals, are mostly seen within the rotator cuff tendons, but rarely, the other tendons around the shoulder (trapezius, pectoralis major, and biceps brachii) are also involved [3–6]. To our knowledge, no case of calcification around the proximal insertion site of the deltoid was reported in the literature. Here, we presented a case of calcification at the insertion site to the anterior acromion of left deltoid, which was successfully treated by conservative management.

## 2. Case Presentation

**Figure 1:**
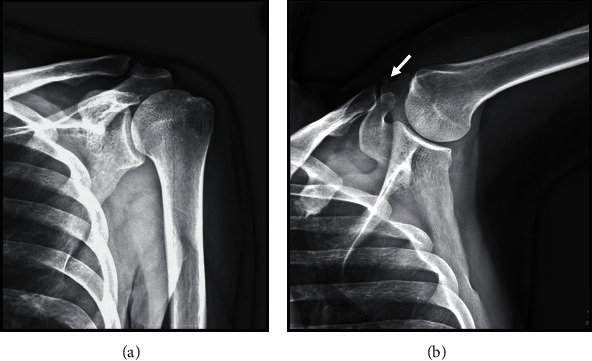
(a) Anteroposterior radiograph of the left shoulder showing a normal appearance. (b) Plain radiograph with the arm in an elevated position showing calcification around the anterior acromion. The arrow indicates the calcium deposit.

**Figure 2:**
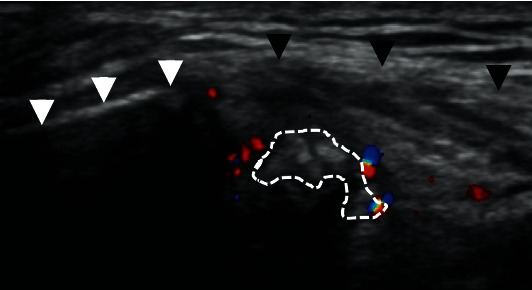
Color Doppler sonogram showing fragmented calcification (dotted line) with hypervascularity, the anterior acromion (white arrowheads), and the deltoid (black arrowheads).

**Figure 3:**
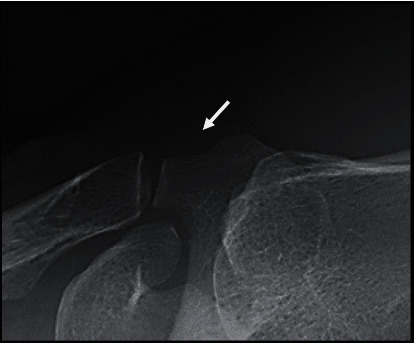
Plain radiograph with the arm in an elevated position showing the disappearance of calcification.

A 45-year-old right-handed man felt a sudden left shoulder pain upon waking up in the morning 2 days before his initial visit to our clinic; later, the pain worsened. He had no clinical history of any metabolic disease or trauma around the shoulder. Physical examination of the left shoulder revealed limited forward flexion to 110°, extension to 30°, abduction to 110°, external rotation to 30°, and internal rotation to L2. The numeral rating scale for pain was 3/10 at rest, 7/10 at motion, and 5/10 at night. Tenderness was noted around the anterior aspect of the acromion. Plain radiography with the left arm in an elevated position confirmed a calcification at the anterior aspect of the acromion, whereas an anteroposterior (AP) radiograph revealed a normal appearance (Figure 1). Ultrasonography revealed a small hyperechoic lesion on the anterior acromial insertion of the left deltoid muscle and hypervascularity around the calcium deposit (Figure 2). Rotator cuff tears and subacromial bursitis were not observed on ultrasonography. The patient received a nonsteroidal anti-inflammatory drug (NSAID) and a local injection of triamcinolone acetonide (15 mg) and 1% lidocaine (3 cm^3^) around the calcification. A few days after medical treatment, the patient's pain ameliorated dramatically with the restoration of a full range of motion. No recurrence of symptoms was noted over the following 3 years. At 3 years after the initial treatment, plain radiographs of the left shoulder demonstrated complete disappearance of the calcification (Figure 3).

## 3. Discussion

The present case report details our experience with anterior deltoid calcification. Calcific tendinitis of the shoulder commonly occurs in the rotator cuff tendons [1]. Although calcium deposits at the distal insertion site of the deltoid have been reported in the literature, those in acromial attachment site of the deltoid have not been reported [7].

Calcific tendinitis of the rotator cuff often has acute symptoms, including severe pain, tenderness, local edema and swelling, restrictive active and passive range of motion, and mild fever, which are partly caused by subacromial bursitis due to extrusion of the calcium deposit. Similarly, the present case had progressively increasing acute pain for 2 days without any trauma. Symptoms of deltoid calcification could be hard to differentiate from those of calcific tendinitis of the rotator cuff. Calcification in this case was confirmed by radiography with the arm in an elevated position, but not in the common anteroposterior (AP) view. Because calcification at the deltoid could be hidden in the acromion or clavicle when the AP view on X-rays was taken, directions other than AP view might be useful, such as scapular-Y or the arm in an elevated position.

Calcium deposits had also been detected on ultrasonography. Some reports have suggested the utility of ultrasonography in the diagnosis of calcific tendinitis of the shoulder [8, 9]. Chiou et al. classified the shapes of calcific plaques into four groups: arc, fragmented/punctate, nodular, and cystic, and suggested that plaques appearing fragmented, nodular, or cystic type should be considered in the resorptive phase [9]. Also, the nonarc-shaped group exhibited more hypervascularity and pain than the arc-shaped group. In this case, the resorptive phase of fragmented calcium deposits with hypervascularity preceded complete resorption.

The patient needs conservative treatment as natural resorption (healing) is expectant for this pathology [10]. The first treatment of choice should include rest and NSAIDs. However, other therapeutic options such as steroid injections, ultrasound- (US-) guided percutaneous aspiration, or extracorporeal shock wave therapy should be considered [11–13]. NSAID use and US-guided local injection of triamcinolone acetonide (a synthetic corticosteroid) dramatically decreased the patient's shoulder pain in the next days.

## 4. Conclusion

The present case report highlights our experience with deltoid tendon calcification. As a differential diagnosis, acromioclavicular joint disorder, subdeltoid bursitis, or calcification of coracoacromial ligament should be considered. Patients presenting with acute shoulder pain without any trauma will be suspected by clinicians to have calcific rotator cuff tendinitis. However, in the absence of calcification around the rotator cuff, clinicians should carefully palpate other sites such as the deltoid, long head of biceps, pectoralis major, and trapezius and examine using ultrasonography or radiography as well.
